# Plant Immune Elicitors for Postharvest Fruit Preservation: Multifunctional Benefits, Emerging Secreted Protein Elicitors, and Future Perspectives

**DOI:** 10.3390/ijms27177699

**Published:** 2026-08-28

**Authors:** Donghai Xie, Chan Xu, Juanni Yao, Yulin Cheng

**Affiliations:** Key Laboratory of Plant Hormones and Molecular Breeding of Chongqing, School of Life Sciences, Chongqing University, Chongqing 401331, China

**Keywords:** postharvest fruit diseases, plant immune elicitors, secreted proteins, fruit quality, artificial intelligence

## Abstract

Fruits contain abundant diverse nutrients and are an essential component of the human diet. Postharvest pathogens cause severe fruit decay and even mycotoxin contamination, leading to enormous economic losses and food safety risks worldwide. Plants have evolved an intricate immune system to counter pathogen attacks, and plant immune elicitors, particularly biogenic elicitors, offer a promising approach for eco-friendly plant disease management. Although mechanistic studies of plant immunity have largely focused on leaf tissues, considerable progress has been made in the application of immune elicitors for controlling postharvest fruit diseases. In this review, we categorize the main types of plant immune elicitors for postharvest disease control, and highlight that certain elicitors possess multifunctional benefits, including improving fruit quality and enhancing fruit abiotic stress tolerance. Meanwhile, we summarize the molecular mechanisms of newly identified secreted protein elicitors. Finally, we outline a roadmap that combines high-throughput screening with artificial intelligence to accelerate the discovery of novel secreted protein elicitors and their plant receptors, while critically assessing translational barriers to their commercial application in fruit preservation.

## 1. Introduction

Fresh fruits are important sources of vitamins, dietary fiber, and water and thus form an essential part of the human diet. As a major component of global agricultural trade, fruits contribute to economic growth in the agricultural sector and enrich diets worldwide. However, severe fruit losses during postharvest storage and transportation cause substantial annual economic losses [1,2]. In addition to fruit ripening and senescence, postharvest diseases caused by pathogenic fungi are a major cause of fruit decay [3]. *Botrytis cinerea* is a destructive, broad-host-range necrotrophic phytopathogen that causes gray mold and fruit rot at both preharvest and postharvest stages [4]. In addition, *Penicillium expansum* [5], *P. digitatum* [1,6], and *P. italicum* [7] are notorious postharvest fungal pathogens of fruit. *P. digitatum* accounts for up to 90% of postharvest losses in citrus fruit in subtropical regions [1]. Notably, most postharvest pathogens are necrotrophs. Moreover, some postharvest fungal pathogens produce mycotoxins such as patulin and ochratoxin, posing serious risks to consumer safety [8].

For decades, synthetic fungicides have been the primary strategy for controlling postharvest fruit diseases [2]. However, their prolonged and widespread use has promoted fungicide resistance in pathogenic fungi, creating a vicious cycle of increasing application rates and resistance [9]. Excessive pesticide residues can also create trade barriers for fruit and vegetable exports [10]. Driven by global initiatives to reduce pesticide use and growing consumer demand for food safety, new technologies for controlling plant diseases, including postharvest fruit diseases, are urgently required in global agriculture [11].

Understanding the molecular mechanisms of plant immunity is fundamental to the development of effective disease-control strategies [12]. Plant cell-surface and intracellular receptors recognize diverse molecules and establish pattern-triggered immunity (PTI) and effector-triggered immunity (ETI) [13,14]. PTI relies on cell-surface pattern-recognition receptors (PRRs) that recognize pathogen-associated molecular patterns (PAMPs) or damage-associated molecular patterns (DAMPs) [15], and it represents the primary line of defense against necrotrophic pathogens [16]. Detailed study of PRR evolution helps elucidate the molecular mechanisms of PTI and provides a foundation for receptor engineering [15].

Plant immune elicitors activate plant immunity and enhance disease resistance [17]. This field has developed rapidly, and findings from basic research have been translated into field applications [11]. Biogenic elicitors represent a promising strategy for eco-friendly plant disease management [11]. Since harpin, a protein that triggers hypersensitive responses, was identified in *Erwinia amylovora*, the field of protein elicitors has developed rapidly [18]. Beyond microbial elicitors, insect secretory proteins also possess elicitor functions [19]. The oral secretory protein GSS1 and its core peptide GSS1-P1 from *Plutella xylostella* induce endogenous abscisic acid accumulation in the host and thereby enhance plant resistance to insects [20]. Most mechanistic studies of plant immune elicitors remain centered on leaf immune networks [21] and model plants [12]. Moreover, fruit resistance is shaped by fruit physiology, maturation status, and host–pathogen interactions [22]. However, an integrated review that jointly examines postharvest disease control, fruit quality, and abiotic stress tolerance remains lacking.

This review presents an integrated framework connecting immune-elicitor classification, multifunctional benefits, secreted-protein mechanisms, and artificial intelligence (AI)-assisted screening. Specifically, we searched Google Scholar and Web of Science using the keywords “fruit”, “elicitor”, “immunity” and “disease” and covering publications from 1990 to the present. Rather than focusing only on disease suppression, we examine the broader roles of immune elicitors in postharvest preservation and identify the key gaps that must be addressed before secreted protein elicitors are translated into commercial products.

## 2. Types of Postharvest Fruit Immune Elicitors

**Figure 1:**
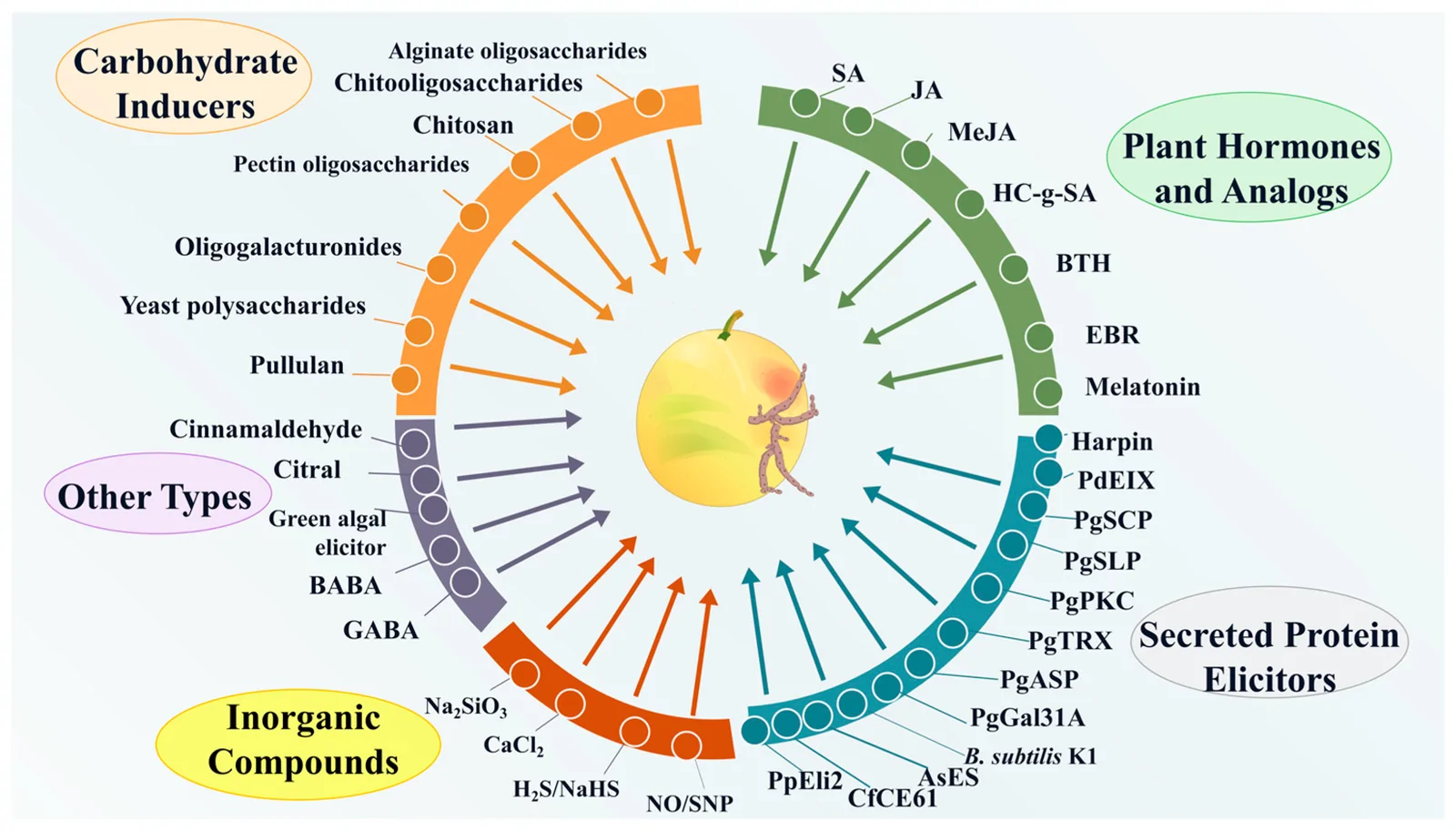
Major categories of postharvest fruit immune elicitors. Different colors represent different types of elicitors. SA: salicylic acid; JA: jasmonic acid; MeJA: methyl jasmonate; HC-g-SA: hemicellulose-grafted salicylic acid; BTH: benzothiadiazole; EBR: 24-epibrassinolide; BABA: β-aminobutyric acid; GABA: γ-aminobutyric acid; H_2_S: hydrogen sulfide; NaHS: sodium hydrosulfide; NO: nitric oxide; SNP: sodium nitroprusside; Na_2_SiO_3_: sodium metasilicate; CaCl_2_: calcium chloride.

Based on their origins, elicitors of postharvest fruit immunity are broadly classified into five categories: carbohydrate elicitors, phytohormones and their functional analogs, secreted protein elicitors, inorganic compounds, and other elicitor types (Figure 1).

### 2.1. Carbohydrate Elicitors

The potential of carbohydrates as agricultural plant immune elicitors has recently been reviewed systematically [23]. Carbohydrate elicitors typically activate downstream defense responses through plant immune signaling. These responses include reactive oxygen species (ROS) accumulation, enhanced defense-related enzyme activity, metabolic reprogramming, wound healing, and cell-wall reinforcement, thereby improving plant resistance to biotic stress. Carbohydrate elicitors, including chitosan, chitooligosaccharides, oligogalacturonides, alginate oligosaccharides, pectic oligosaccharides, yeast saccharides, and pullulan, have been widely studied in postharvest fruit.

Chitosan is a carbohydrate elicitor that has been extensively studied in postharvest fruit. Chitosan combines elicitor, antimicrobial, and film-forming properties [24]. A meta-analysis indicates that its antimicrobial and eliciting activities coexist [25]. Chitosan induces disease resistance and regulates ROS metabolism in navel oranges [26]. The combination of chitosan and *Pichia membranaefaciens* promotes wound healing in citrus as a resistance mechanism against *Colletotrichum gloeosporioides* [27]. This treatment also suppresses anthracnose by maintaining cell structural integrity in citrus fruit [28]. Chitosan reduces postharvest decay and induces defense responses in kiwifruit [29]. Low-molecular-weight chitosan suppresses decay caused by *Botryosphaeria* sp. and preserves pear quality [30]. Chitosan induces jasmonic acid (JA) production in postharvest strawberries and grapes, contributing to enhances resistance to gray mold [31]. Chitosan also inhibits *P. digitatum* and *P. italicum* and protects citrus fruit from green and blue molds during postharvest storage [32].

Pectic oligosaccharide (POS) treatment reduces postharvest decay in kiwifruit, including blue mold caused by *P*. *expansum* and black rot caused by *Alternaria alternata* [33]. The mechanism involves increased expression and activity of chitinase, β-1,3-glucanase, and superoxide dismutase. Oligogalacturonides (OGs) accelerate mechanical wound healing in tomato fruit and reduce infection by *B*. *cinerea*. This process depends on a Ca^2+^-mediated systemic acquired resistance (SAR)-related defense pathway [34]. The combination of *Meyerozyma guilliermondii* and alginate oligosaccharide controls citrus blue mold [35], and alginate oligosaccharide also enhances the biocontrol activity of *Debaryomyces hansenii* against postharvest apple blue mold [36]. Agaro-oligosaccharides induce peach resistance through JA signaling involving PpMYC2-activated *PpPGIP1* expression [37]. Yeast saccharide inhibits postharvest *P*. *expansum* decay by inducing endogenous nitric oxide (NO) accumulation in peach fruit and enhancing defense-related enzymes, including chitinase, β-1,3-glucanase, phenylalanine ammonia-lyase, and peroxidase [38].

Pullulan coating combined with calcium chloride (CaCl_2_) dipping modulates brown spot development during cold storage in ‘Huangguan’ pear [39]. When loaded with biocontrol yeast and sodium chloride (NaCl), a pullulan-based coating inhibits postharvest decay in citrus fruit [40]. Pullulan also induces resistance to *Diaporthe nobilis* in postharvest kiwifruit [41]. Fungal cell-wall oligosaccharides, including β-1,3-glucan, trigger plant immune responses as microbe-associated molecular pattern (MAMP)-like signals [23,42]. β-1,3-glucan treatment induces phytoalexin accumulation and enhances resistance to gray mold [43]. In contrast, α-1,3-glucan interferes with plant innate immunity and promotes latent fungal infection [44]. Green-algae-derived ulvan and oligoulvan induce defense responses against *P. expansum* and *B. cinerea* in apple fruit [45]. Other saccharides, including dextran [46] and yeast saccharides [38], have also been applied in immune-based management of postharvest fruit diseases.

### 2.2. Plant Hormones and Functional Analogs

Plant hormones and their functional analogs regulate immune processes, including pathogenesis-related (PR) protein expression and systemic acquired resistance (SAR), by coordinating endogenous hormone signaling and thereby enhancing postharvest fruit defenses against pathogen infection [47,48]. JA and salicylic acid (SA) are two core hormones in plant immune regulation. Their roles in plant immunity have been reviewed [49]. JA primarily mediates responses to necrotrophic pathogens, herbivores, and wounding [48]. SA mainly promotes local defense and systemic acquired resistance against biotrophic and hemibiotrophic pathogens [49]. Their crosstalk is antagonistic or synergistic depending on pathogen lifestyle, tissue context, and signal intensity, thereby fine-tuning defense allocation [49].

Methyl jasmonate (MeJA) treatment increases tomato fruit resistance to *B. cinerea* through the JA signaling pathway [50], and this response depends on the key transcription factor SlMYC2 [51,52]. MeJA also enhances sweet cherry resistance to *Monilinia fructicola* and increases the activities of defense-related enzymes, including β-1,3-glucanase, phenylalanine ammonia-lyase (PAL), and peroxidase (POD) [53]. In grape berries, MeJA-induced resistance to gray mold is concentration-dependent and is associated with hydrogen peroxide (H_2_O_2_) production, increased *VvNPR1.1* expression, and phytoalexin accumulation [54].

SA treatment enhances resistance to *B. cinerea* in tomato plants [55]. In sweet cherry, SA reduces *P. expansum* infection by increasing the activities of antioxidant and PR-related enzymes, including catalase (CAT), glutathione peroxidase (GPX), chitinase, and β-1,3-glucanase [56]. SA treatment also reduces postharvest citrus decay, and its effect is associated with the accumulation of defense-related metabolites, including H_2_O_2_, ornithine, threonine, and lipophilic polymethoxylated flavones in the peel [57]. Transcriptomic and biochemical analyses show that SA, *P. membranaefaciens*, and oligochitosan treatments significantly induce phenylpropanoid metabolism in citrus [58]. In blueberry, SA-grafted bamboo hemicellulose inhibits *B. cinerea* infection and alters the phenylpropanoid pathway and peel microbial community [59]. The combination of an SA analog, cinnamon oil (whose main component is cinnamaldehyde), and a *Bacillus* antagonist also induces systemic resistance to anthracnose in mango [60].

Benzothiadiazole (BTH) is a synthetic SA analog that efficiently activates the SAR pathway [61]. Postharvest BTH treatment increases peach resistance to *P. expansum* by activating defense enzymes, including PAL, POD, chitinase, and β-1,3-glucanase [62]. BTH-induced defense against mango anthracnose is associated with *polyphenol oxidase* (*PPO*) and *POD* gene expression and polyphenol accumulation [63]. BTH treatment also induces banana resistance to anthracnose and activates resistance-related genes such as *PR1* and *PTI5* [64]. These findings indicate that diverse hormone-related compounds and microbial antagonists support postharvest disease management.

Melatonin enhances tomato resistance to *B. cinerea* by activating the JA signaling pathway [65]. However, in citrus, melatonin reduces fruit resistance by excessively scavenging defense-related ROS [66]. As a brassinosteroid, 24-epibrassinolide improves peach resistance to *Rhizopus stolonifer* by modulating cell-wall polysaccharide metabolism [67]. 24-epibrassinolide (EBR) also reduces postharvest disease incidence and induces stress-related gene expression in *Satsuma mandarin* [68].

### 2.3. Secreted Protein Elicitors

Protein immune elicitors are proteins derived from pathogenic microorganisms, antagonistic microorganisms, or plants. Host recognition of these protein signals usually initiates PTI-related immune responses, defense-enzyme activation, secondary metabolic reprogramming, and cell-wall reinforcement. However, the pattern-recognition receptors and co-receptor binding sites of most secreted protein elicitors in fruit remain unknown.

Harpin was originally identified in *Erwinia amylovora* as a protein elicitor that induces the hypersensitive response [18]. Preharvest spraying or postharvest treatment with harpin reduces blue mold caused by *P*. *expansum* in apple fruit [69]. In postharvest Hami melon and muskmelon, harpin enhances resistance to *Trichothecium roseum* by increasing POD and chitinase activities [70,71]. Treatment with the commercial harpin preparation Messenger reduces citrus black spot in sweet orange fruit [72]. Harpin also enhances kiwifruit resistance to *B. cinerea* and *P. expansum* by increasing PPO, POD, and superoxide dismutase (SOD) activities and promoting lignin accumulation [73]. In tomato fruit, it enhances resistance to *B. cinerea* and *Alternaria alternata* by upregulating genes related to chitinase, β-1,3-glucanase, and PAL, increasing the corresponding enzyme activities, and promoting total phenol and lignin accumulation [74].

In fresh jujube stored at 4 °C for 28 d, 30 mg/L harpin reduced the disease index by approximately 54%, reduced weight loss by approximately 38%, and increased firmness by approximately 123% relative to the control [75]. In harvested Hami melons, harpin treatment at 90 mg/L induced local and systemic resistance to *Trichothecium roseum* [70]. Suppression of lesion development persisted for 8 d in harpin-treated fruit and 6 d in their untreated counterparts in the ‘8601’ cultivar [70]. The corresponding durations in the ‘New Queen’ cultivar were 5 and 3 d [70]. Microscopy further showed that, in untreated apple wounds, *P. expansum* colonization was evident within 48 h and tissue penetration occurred by 72 h, whereas harpin-treated fruit showed no evident spore germination or wound colonization until 96 h and colonization was observed only at 144 h [76].

PdEIX from *P. digitatum* functions as a PAMP that enhances resistance to fungal pathogens in several fruit systems [77]. Secreted proteins from *Bacillus subtilis* K1 (GM001344) induce apple resistance to *B. cinerea* [78]. Pretreatment with GM001344 24 h before the *B. cinerea* challenge reduced disease incidence from 100% to 63.5% and the disease severity index from 76.75 to 38.43 [78]. After 6 d, treated fruit also retained significantly higher firmness, total soluble solids, and ascorbic acid than controls [78].

The *Pythium periplocum* elicitin PpEli2 is recognized by the cell-surface receptor-like protein REli and promotes interaction between REli and the co-receptor BAK1, thereby triggering receptor-dependent immune responses and improving broad-spectrum resistance to oomycete and fungal diseases in several plants [79]. The heat-stable elicitor CfCE61, secreted by *C. fructicola*, interacts with PbrPOD1 and enhances pear resistance to bitter rot [80]. The *Acremonium strictum*-derived elicitor AsES induces ethylene synthesis in strawberry fruit and reduces gray mold caused by *B. cinerea* [81]. Inhibition of ethylene perception by 1-methylcyclopropene (1-MCP) weakens this protective effect, indicating that AsES-induced resistance is associated with ethylene-mediated defense [81]. Few studies have examined plant-derived secreted proteins that induce postharvest fruit immunity and enhance disease resistance. LcPIP1 interacts with the pectate lyases PlPeL1 and PlPeL1-like from *Peronophythora litchii* [82]. Exogenously synthesized LcPIP1 enhances postharvest litchi resistance to *P. litchii*, and LcWRKY34 promotes fruit resistance by activating *LcprePIP1* expression [83].

Several secreted proteins from *Pichia galeiformis* protect citrus fruit through distinct metabolic pathways. PgSLP activates amino acid metabolism [84], and PgPKC promotes valine biosynthesis [85]. PgTRX is a thioredoxin-like protein elicitor associated with glutathione metabolism [86]. PgASP is associated with phenylalanine and tyrosine metabolism [87]. Moreover, PgGal31A inhibits citrus blue mold by altering cell-wall components [88].

### 2.4. Inorganic Compounds

Silicates, carbonates, bicarbonates, and calcium salts are representative inorganic compounds. These compounds can be used for postharvest cleaning, soaking, fumigation, or storage treatments. Sodium silicate treatment reduces postharvest decay in Hami melon by inducing the production of resistance-related peroxidase and chitinase, and inhibiting fungi [89]. Sodium carbonate and sodium bicarbonate induce resistance to postharvest green mold in citrus fruit [90].

Calcium chloride (CaCl_2_) has been widely studied as an inorganic elicitor of postharvest disease resistance in fruit. In kiwifruit, CaCl_2_ induces defense and respiratory burst oxidase homolog (*RBOH*) gene expression, promotes ROS accumulation, and increases antioxidant enzyme activity, thereby enhancing resistance to *Diaporthe nobilis* [91]. In peach fruit, CaCl_2_ pretreatment elicits defense responses and JA signaling, promotes ROS and pathogenesis-related protein accumulation, and thereby enhances resistance to *Monilinia fructicola* [92]. Preharvest calcium fertilizer application significantly reduces postharvest apple brown rot caused by *M*. *fructigena* [93].

Nitric oxide (NO) is well established as a postharvest regulator of fruit immune responses [94,95]. The nitric oxide donor sodium nitroprusside (SNP) activates antioxidant defenses, promotes the shikimate pathway and phenylpropanoid metabolism, increases the accumulation of phenols, flavonoids, and lignin, and enhances the resistance of apples to *A. alternata* after harvest [96]. In pitaya fruit, exogenous NO treatment enhances postharvest defense responses and reduces anthracnose development [97]. NO treatment also enhances peach resistance to *M. fructicola* by activating the γ-aminobutyric acid (GABA) shunt [98]. Hydrogen sulfide (H_2_S) donors such as sodium hydrosulfide can also induce fruit resistance. In apple, sodium hydrosulfide activates phenylpropanoid metabolism and lignin biosynthesis through hydrogen peroxide- and nitric oxide-mediated signaling pathways and enhances resistance to *P. expansum* [99].

### 2.5. Other Types of Elicitors

Other elicitors discussed here include amino acids and their derivatives, oxalic acid, glycerol, ozone, cinnamaldehyde, citral, and natural biostimulants. Their disease control functions typically include inducing host defense as well as direct antibacterial, bactericidal, or antifungal activity.

Ozone treatment induces plant defense enzymes and regulates ethylene biosynthesis in jackfruit, thereby controlling fruit rot and maintaining postharvest quality [100]. Cinnamaldehyde treatment promotes defense responses in postharvest citrus fruit inoculated with *P. digitatum* and *Geotrichum citri-aurantii* [101]. Citral treatment enhances disease resistance in postharvest citrus fruit by activating the JA pathway and promoting phenylpropanoid accumulation [102]. Natural biostimulants have also been incorporated into integrated management of postharvest citrus green mold and stimulate citrus immunity [103].

Amino acids and their derivatives exhibit immune-eliciting activity. In red pitaya, GABA-induced disease resistance involves the mitogen-activated protein kinase (MAPK) cascade and ROS metabolism [104]. In tomato fruit, GABA triggers defense against *B. cinerea* by regulating ethylene and JA signaling [105] and enhances resistance to *A. alternata* [106]. In mango, GABA treatment enhances resistance to *C. gloeosporioides* [107]. β-Aminobutyric acid (BABA) induces resistance to *B. cinerea* in tomato fruit [108] and has also been evaluated for mango anthracnose management [109]. In Guifei mango inoculated with *C. gloeosporioides*, 50 mmol/L BABA was evaluated after 15 d: all control fruit developed disease, with a mean lesion diameter of 36.58 mm, whereas BABA-treated fruit showed 60–76.67% incidence and 13.37–22.75 mm lesions, corresponding to 36.55–62.19% of the control lesion diameter [109]. The BABA receptor PpIBI1 integrates early PTI and late SAR responses to enhance postharvest peach resistance [110].

Overall, postharvest fruit immune elicitors are diverse and differ in origin, chemical properties, modes of action, and application potential. Table 1 summarizes and compares four major elicitor classes: carbohydrate elicitors, plant hormones and their functional analogues, secreted protein elicitors, and inorganic compounds. The comparison focuses on their main mechanism, advantages, and limitations. Other elicitors are not included in Table 1 because their chemical properties, targets, and disease-control modes are too heterogeneous to be compared within a unified framework.

## 3. Specific Action Mechanisms of Emerging Secreted Protein Elicitors

**Figure 2:**
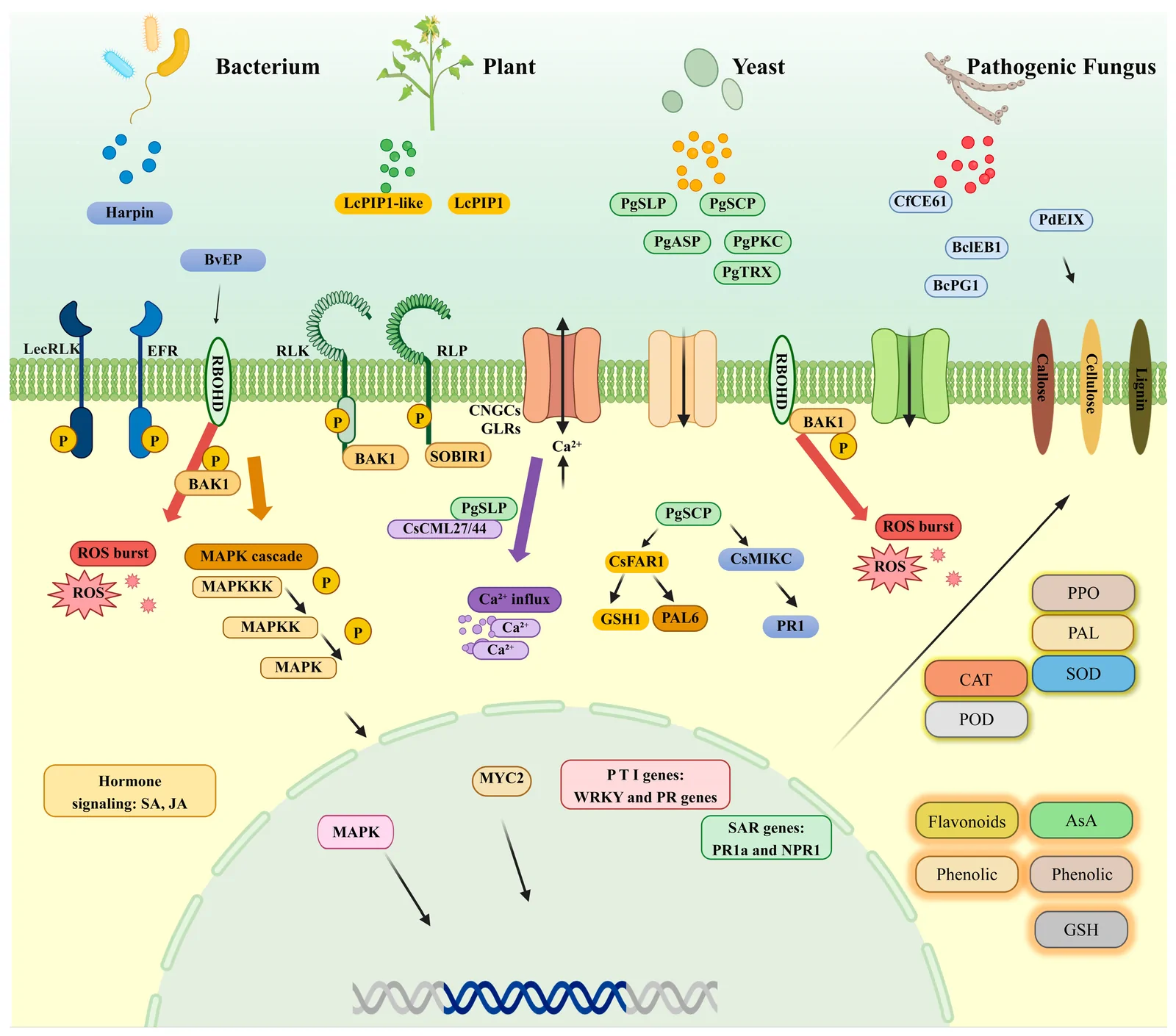
Model of secreted protein elicitor-mediated fruit immunity. Secreted protein elicitors from pathogenic fungi, yeasts, plants, and bacteria activate fruit immune signaling through cell-surface receptor recognition or intracellular interactions and subsequently induce transcriptional and metabolic reprogramming. Downstream defense responses include pathogenesis-related protein accumulation, antioxidant enzyme activation, antimicrobial metabolite production, callose deposition, and lignification, which ultimately enhance fruit resistance to postharvest diseases. RLK: receptor-like kinase; RLP: receptor-like protein; ROS: reactive oxygen species; MAPK: mitogen-activated protein kinase; SA: salicylic acid; JA: jasmonic acid; PTI: pattern-triggered immunity; WRKY: WRKY transcription factor family; PR genes: pathogenesis-related genes; SAR: systemic acquired resistance; PPO: polyphenol oxidase; PAL: phenylalanine ammonia-lyase; CAT: catalase; SOD: superoxide dismutase; POD: peroxidase; AsA: ascorbic acid; GSH: glutathione; P: phosphorylation.

Secreted protein elicitors activate host defenses at multiple levels and thereby enhance postharvest fruit disease resistance. The specific action mechanisms involve cell-surface recognition, early immune signaling, host-protein interactions, and downstream transcriptional and metabolic regulation (Figure 2).

### 3.1. Early Signals: ROS, Ca^2+^, and MAPK

Upon recognition by receptor-like kinases (RLKs) or receptor-like proteins (RLPs), exogenous protein signals initiate Ca^2+^ influx, ROS bursts, and protein kinase phosphorylation cascades through co-receptors or signaling components such as BAK1 and SOBIR1. These events induce defense gene expression and disease resistance [115]. ROS bursts are the most commonly detected early defense signals [116]. Harpin treatment increases H_2_O_2_ levels in jujube fruit [75]. PpEli2 induces H_2_O_2_ accumulation in *Nicotiana benthamiana*, tomato, pepper, and soybean leaves [79]. Secreted proteins from *B*. *subtilis* K1 increase POD and CAT activities and reduce malondialdehyde (MDA) accumulation in apple fruit [78]. Ca^2+^ signaling connects extracellular sensing with intracellular responses, and PgSLP interacts with citrus calmodulin-like proteins CsCML27 and CsCML44 to improve fruit disease resistance [117]. Together, these studies indicate that secreted protein elicitors link early recognition to metabolic and defense responses through ROS, antioxidant enzymes, and calmodulin-like (CML)-mediated Ca^2+^ signaling. The MAPK cascade is a central hub linking early signaling to transcriptional regulation [118]. LcPIP1 treatment increases cytoplasmic Ca^2+^, ROS accumulation, and MAPK activation in litchi fruit [83].

Evidence for receptor recognition is elicitor-specific and is not universal. PdEIX is localized to the apoplast and induces immune responses in citrus fruit [77]. PdEIX-induced cell death in *N. benthamiana* requires the receptor-like protein NbEIX2, the receptor-like kinase BAK1, and other immune-signaling components [77]. PpEli2 is recognized by the receptor-like protein REli, which enhances its interaction with BAK1 [79]. In contrast, LcPIP1 interacts with SERK3, a BAK1 homolog that is essential for LcPIP1-induced cell death in *N. benthamiana* [82]. LcPIP1 also binds the pathogen pectate lyase PlPeL1 and its homologs, weakening their susceptibility-promoting activity [82]. These findings support two distinct modes of elicitor activity: receptor/co-receptor recognition and direct host–pathogen protein interactions.

### 3.2. Transcriptional Reprogramming

Transcriptional reprogramming is a key downstream mechanism by which secreted protein elicitors translate host-recognition signals into disease resistance. Evidence from two studies shows that PgSCP recruits distinct citrus transcription factors. PgSCP interacts with CsMIKC [112]. CsMIKC binds to the promoters of the *PR1-like* and *ATPase* genes and activates their transcription. PgSCP also interacts with CsFAR1 [113], which positively regulates citrus resistance and activates a broader set of defense-related genes, including *DHAPS-1*, *GSH1*, *PAL6*, *CYP73A16*, *CYP73A4*, and *PER16*. Together, these findings indicate that a single elicitor can engage multiple transcription factor-dependent defense pathways. In a related system, LcWRKY34 activated *LcprePIP1* expression during LcPIP1-associated resistance in litchi fruit [83]. Thus, the directly supported transcriptional events comprise elicitor–transcription factor interactions, activation of specific defense-gene promoters or transcripts, and the resulting resistance phenotypes.

### 3.3. Primary and Secondary Metabolic Reprogramming

Durable induced resistance requires the translation of transcriptional reprogramming into metabolic allocation and energy use. Specialized metabolic reprogramming supports defense allocation [119]. Primary metabolism supplies energy for defense, and central carbon and amino acid metabolism links organic acids, energy use, and secondary metabolic pathways [120].

PgSLP enhances amino acid metabolism [84]. PgPKC is associated with increased branched-chain amino acid transaminase activity and valine biosynthesis [85]. In the PgASP study, exogenous phenylalanine and tyrosine were associated with phenol and lignin production and blue-mold resistance [87].

The individual proteins therefore show distinct metabolic readouts. PgSLP and harpin promote glutathione accumulation [75,84], whereas PgASP, PgSCP, and harpin are associated with phenol/lignin accumulation [74,87,113]. Glutathione metabolism provides ROS buffering, detoxification, and reducing power [121]. PgTRX-associated suppression of citrus green mold involves glutathione metabolism [86], while harpin treatment increases glutathione reductase activity and glutathione (GSH) content in winter jujube fruit [75]. Together, these responses contribute to redox homeostasis during induced defense.

Protein-elicitor treatments repeatedly affect phenylpropanoid and related secondary-metabolic pathways [74,84,87,113]. Harpin increases PAL activity, total phenolics, and lignin in tomato fruit [74]. These metabolites can exert antimicrobial or antioxidant effects, while lignin and cell-wall cross-linking precursors contribute to a physical barrier [122].

### 3.4. Reinforcement of Physical Barriers

Strengthening physical barriers is an important pathway through which secreted protein elicitors enhance postharvest fruit resistance. Postharvest pathogens usually infect fruit through microcracks, mechanical wounds, or cell-wall degradation. Therefore, cell-wall remodeling, callose deposition, and lignin accumulation hinder pathogen infection and spread.

Lignin and callose deposition strengthen physical defenses at the cell wall. Harpin enhances lignin accumulation in kiwifruit [73], promotes lignin production in tomato fruit [74], and induces apple cell-wall reinforcement [76]. PdEIX induces callose production in citrus fruit [77]. LcPIP1 treatment promotes lignin accumulation in litchi fruit [83].

## 4. Multifunctional Benefits of Postharvest Fruit Immune Elicitors

The benefits of plant immune elicitors are not limited to postharvest fruit disease control. Some elicitors also maintain fruit quality and improve tolerance to abiotic stress. Reported examples are summarized in Table 2. In addition to enhancing disease resistance, elicitor treatments promote the accumulation of ascorbic acid, anthocyanins, flavonoids, carotenoids, and soluble sugars or delay their consumption and degradation during storage, thereby affecting fruit color and taste. Some immune elicitor treatments also enhance tolerance to chilling or low-temperature stress.

### 4.1. Improvement of Fruit Quality

Ascorbic acid (AsA) is an important water-soluble antioxidant in fruit that limits ROS accumulation. Several elicitor treatments enhance fruit disease resistance while promoting AsA accumulation. Examples include Kadozan-treated litchi [123], winter jujube treated with chitosan and sodium silicate [124], and grapefruit treated with carboxymethyl chitosan [125]. Other examples include papaya and winter jujube treated with melatonin [75,126], winter jujube treated with β-aminobutyric acid [127], citrus treated with sodium nitroprusside [128], and kiwifruit sprayed with oxalic acid [129]. These treatments increase AsA levels or maintain them at higher levels in fruit.

Anthocyanins are flavonoid pigments that impart red, purple, and blue colors to fruit and possess antioxidant activity [130]. MeJA treatment of strawberry fruit [131] and MeJA treatment alone or combined with 1-MCP fumigation in pomegranate fruit [132] promote anthocyanin accumulation. Melatonin treatment of kiwifruit [133] and plum [134] promotes anthocyanin accumulation. POS treatment increases total phenol and anthocyanin contents in strawberry fruit [135], whereas BTH treatment promotes anthocyanin accumulation in strawberry fruit [136].

Fruit flavor is primarily determined by sugars, acids, and volatile compounds. Among postharvest resistance-inducing treatments, BABA maintains a high soluble sugar content in peach fruit [137]. SA inhibits blue mold and maintains higher total soluble solids (TSS) and titratable acidity (TA) [138,139]. Grapes treated with 40 mM GABA retain higher levels of soluble sugars and organic acids [140]. The *B*. *subtilis* K1 secreted protein GM001344 enhances apple TSS [78]. MeJA treatment increases carotenoids, soluble solids, and lycopene in tomato fruit [141,142]. Melatonin reshapes sugar metabolism and transport in postharvest tomato fruit, increases the sugar-to-acid ratio, and promotes lycopene accumulation [143,144].

In postharvest blueberry fruit inoculated with *A. tenuissima*, 1 mM L-arginine lowered the disease index by 32.05% on day 6 compared with the control [145]. In ‘Angeleno’ plums, postharvest application of 0.1 mM melatonin increased total phenols and anthocyanins in the mesocarp by 21% and 58%, respectively [134]. For ‘Sahebi’ grapes stored at 1 °C for 60 d, 40 mM GABA treatment produced 35% less weight loss, 30% less rachis browning, and 63% lower decay incidence than the control, together with 13% lower electrolyte leakage [140]. For mandarins, a chitosan/phenylalanine coating maintained superior nutritional, antioxidant, and sensory quality for up to 12 d at 21 °C and 35 d at 5 °C; compared with the control, total polyphenols were up to 78.98% higher after 35 d of cold storage and total flavonoids were 42.79% higher after 12 d of shelf life [146].

Regarding aroma, MeJA studies showed that ethylene biosynthesis and the AsA-GSH cycle promoted tomato volatile organic compound formation and improved post-ripening aroma [147]. MeJA alleviates chilling injury and restores aroma-lactone release in peach fruit [148]. Chitosan delays changes in strawberry aroma and enhances fruity esters [149,150]. In mandarin, chitosan and phenylalanine coatings enhance volatile-compound retention and improve the aroma profile, particularly during cold storage [146]. For guava, a 600 μmol/L melatonin dip for 2 h at 25 °C delayed visible disease symptoms until day 15, compared with day 11 in the control, and delayed the soluble-solids peak by approximately 5 d [151].

### 4.2. Improvement of Fruit Tolerance to Abiotic Stress

Chilling injury is a physiological disorder that occurs when cold-sensitive fruit is stored below its critical safe temperature but above the freezing point [152]. It is characterized by visible symptoms such as surface browning, pitting, water-soaked lesions, and abnormal ripening and is closely associated with membrane integrity, energy status, cell-wall metabolism, antioxidant systems, and ROS signaling [153]. Melatonin reduces chilling injury, membrane permeability, chilling injury index, and MDA accumulation in postharvest plum fruit [154]. It also alleviates mango chilling injury by maintaining intracellular energy supply and cell-wall and membrane stability [155]. In ‘Langra’ mango, 100 μM melatonin before storage at 5 °C for 28 d reduced chilling injury from 21% in the control to 5%, whereas ‘Gulab Jamun’ mango showed only a 2-percentage-point reduction, from 38% to 36%, indicating marked cultivar dependence [155]. Progesterone has similar effects [156]. MeJA reduces visible chilling injury in mango [157] and enhances antioxidant enzyme activity in loquat [158]. SA reduces chilling injury, electrolyte leakage, and MDA accumulation [159] and maintains membrane integrity in cold-stored tomato fruit [160]. EBR reduces postharvest disease incidence in citrus, induces stress-related gene expression, and limits membrane damage [68,161].

In blood orange, pretreatment with 10 μmol/L 24-epibrassinolide followed by storage at 5 °C for 42 d and 2 d at 20 °C largely maintained citric acid and ascorbic acid; in controls, citric acid, malic acid, and succinic acid decreased by 32–35%, while ascorbic acid decreased by 70% [161]. For ‘Midknight’ Valencia orange, a 1-min dip in 0.25 mM MeJA prevented visible chilling injury after 90 d at either 4 or 7 °C plus 10 d under simulated shelf conditions, although 0.25 mM and 0.50 mM treatments also reduced soluble solids, titratable acidity, vitamin C, and total antioxidants [162].

**Table 2:** Reported multifunctional benefits of postharvest fruit immune elicitors.

Elicitor Treatment	Fruit	Benefit	References
Pullulan	Kiwifruit	Maintain AsA	[41]
Kadozan chitosan	Litchi	Increase AsA; maintain redox	[123]
Chitosan + sodium silicate	Winter jujube	Increase AsA	[124]
Pectic-oligosaccharides	Strawberry	Promote anthocyanins	[135]
SA	Plum	Mitigate chilling injury	[159]
	Tomato	Mitigate chilling injury	[160,163]
Acibenzolar-S-methyl	Apple	Increase TSS; maintain quality	[164]
BTH	Strawberry	Promote anthocyanins	[136,165]
MeJA	Tomato	Increase AsA and volatile organic compounds (VOCs); promote aroma	[141,147]
	Pomegranate	Mitigate chilling injury; promote anthocyanins	[132,166]
	Peach	Mitigate chilling injury; restore aroma lactones	[148]
	Mango	Mitigate chilling injury	[157,167]
	Loquat	Mitigate chilling injury; modulate AsA-GSH	[158,168]
	Citrus	Mitigate chilling injury	[162]
	Kiwifruit	Mitigate chilling injury	[169]
Melatonin	Winter jujube	Increase AsA	[75]
	Papaya	Increase AsA	[126]
	Plum	Mitigate chilling injury; promote anthocyanins	[134,154]
	Tomato	Modulate sugar; optimize sugar-to-acid	[143,144]
	Litchi	Promote anthocyanins	[170,171]
	Guava	Maintain AsA	[151]
2,4-epibrassinolide	Citrus	Increase sugars and anthocyanins; mitigate chilling injury	[161]
	Pomegranate	Mitigate chilling injury	[172]
	Peach	Mitigate chilling injury	[173,174]
	Blueberry	Maintain soluble solids content (SSC) and TA	[175]
*B. subtilis* K1	Apple	Maintain TSS and AsA	[78]
Harpin	Winter jujube	Increase AsA	[75]
Oxalic acid	Kiwifruit	Increase AsA; delay AsA loss	[129]
NO	Mango	Mitigate chilling injury; delay ripening	[176]
	Plum	Mitigate chilling injury; delay ripening	[177]
	Citrus	Increase AsA; maintain TA and TSS	[128]
Sodium nitroprusside	Peach	Mitigate chilling injury	[178]
GABA	Grape	Maintain soluble sugars and organic acids; mitigate chilling injury	[140]
	Carambola	Increase AsA; mitigate chilling injury	[179]
β-aminobutyric acid	Peach	Maintain soluble sugars; modulate sucrose	[137]
	Pepper	Reduce decay; mitigate chilling injury	[180]
L-arginine	Blueberry	Reduce disease index; promote anthocyanins	[145]
Trans-2-hexenal	Citrus	Increase AsA; maintain AsA-GSH	[181]
Glycerol	Kiwifruit	Delay the decline of SSC and AsA	[182]

## 5. Novel Secreted Protein Elicitors: Discovery and Development Directions

**Figure 3:**
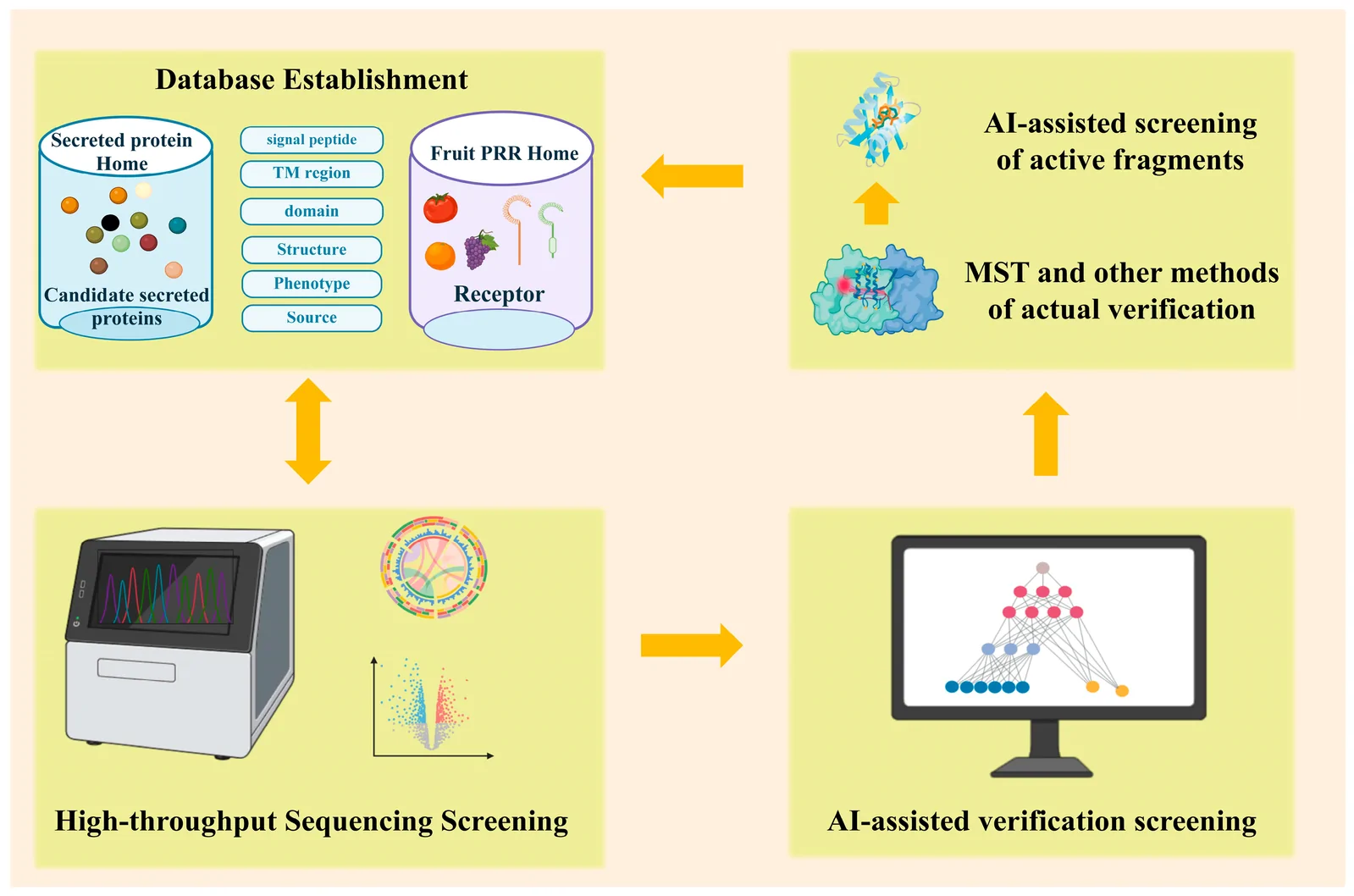
Discovery and development process for secreted protein elicitors used to control postharvest fruit diseases. The process first constructs a dual-candidate database of secreted protein elicitors and fruit cell-surface receptors. High-throughput sequencing data identify candidate secreted proteins that are highly expressed during pathogen infection or elicitor-induced defense and receptors enriched at the fruit surface. AI-assisted pairing predictions then rank candidate protein–receptor interactions for verification. AI-assisted screening also identifies active fragments, converting full-length proteins into smaller active peptides and thereby improving production feasibility and commercial potential. Verified proteins and peptides return to the database for homologous comparison, evidence accumulation, and iterative improvement of prediction reliability. PRR: pattern recognition receptors; TM region: transmembrane region; AI-assisted: artificial intelligence-assisted; MST: microscale thermophoresis. Created in BioRender. XX, X. (2026) https://BioRender.com/f9dh95r, accessed on 31 July 2026.

A core limitation in this field lies not only in the large number of putative secreted proteins but also in the weak evidence chain linking secreted proteins, fruit receptors, functional phenotypes, and application data. Many candidate molecules have been verified only in leaves or heterologous expression systems, and their corresponding receptors remain unclear. AI and structural bioinformatics integrate sequence, expression, structure, and functional information to prioritize elicitor–receptor candidates. However, prediction uncertainties and model biases should be clearly reported, and each candidate pair requires experimental validation. Figure 3 outlines a research framework integrating dual-database construction, candidate prioritization, hierarchical verification, active-peptide development, formulation optimization, and combined application.

### 5.1. Discovery of Novel Secreted Protein Elicitors

First, a ‘Secreted Protein Home’ database should be constructed to include predicted and experimentally verified secreted proteins, with confirmed elicitors clearly labeled. A ‘Fruit PRR Home’ database should be constructed simultaneously to include candidate fruit receptors, including RLKs, RLPs, LysM receptors, LecRLKs, BAK1, SOBIR1, and other co-receptors. Each record should include the signal peptide, transmembrane region, species of origin, expression pattern during infection, experimental evidence, functional domain, version information, and data source and should clearly distinguish predicted from experimentally verified secreted proteins.

On this basis, high-throughput sequencing data may be integrated into the database-based prediction process. Large-scale AlphaFold 3 structural prediction may serve as a hypothesis-generation layer for effector-receptor prioritization, but confidence indices and failure-mode descriptions should be retained [183]. ColabFold provides rapid structural screening [184], and ClusPro molecular docking further screens top-ranked models to identify candidate effector-receptor pairs for subsequent biochemical verification [185]. Rapid screening may use transient expression in *N. benthamiana* to identify high-confidence candidate elicitors. Subsequently, microscale thermophoresis (MST) [186], surface plasmon resonance (SPR) [187], and biolayer interferometry (BLI) [188] may quantify binding affinity, kinetic parameters, and competitive interactions.

Phylogenetic analysis of cell-surface receptors across 350 plant genomes has elucidated the origin, expansion, and differentiation of PRR, RLK, and RLP receptor families [189]. The receptor SCORE from *Citrus maxima* was subsequently identified among LRR-RLK-XII receptors from 285 plant species, and structural prediction and receptor engineering expanded its recognition of cold shock protein (CSP) peptides from different pathogens [190]. These studies demonstrate the feasibility of receptor-resource mining, candidate-receptor screening, and receptor engineering and provide a foundation for constructing the Fruit PRR Home candidate database.

### 5.2. Development Directions for Novel Secreted Protein Elicitors

Production cost remains a major constraint on the industrialization of protein elicitors. Converting full-length proteins into small active peptides may reduce production and quality-control costs. The 35-amino-acid peptide ieb35 from BcIEB1 retained the activity of the original protein [114], indicating that protein elicitors contain separable active fragments. Identification of the minimal active fragment requires truncation analysis, overlapping-peptide screening, key-site mutation, and dose–response experiments.

Protein-elicitor formulations and delivery systems require systematic verification. Processing conditions in aqueous solutions may affect peptide stability [191], and proteins may aggregate or form particles at interfaces [192]. Generic formulations should therefore account for stability throughout processing, production, storage, and transportation [193]. The application of SA microcapsules (SAM) to apple fruit provides a case study comparing SA with SAM. SAM showed control efficacy against postharvest blue mold in apple fruit and helped maintain fruit quality [139]. Chitosan nanocarriers enhance the stability and field performance of the protein elicitor fTDP [194]. The modified membrane-translocation domain MTD4 enables efficient systemic delivery of functional proteins in plants, and MTD4-HrpZ induces stronger SAR in tobacco and tomato [195]. A packinghouse-style citrus assay showed that combining a biostimulant with half-dose imazalil achieved green-mold control comparable to full-dose imazalil, while imazalil residue in the peel was 0.612 ppm, below the 4 ppm European Union threshold for citrus fruit [103].

Combined application strategies for protein elicitors also require investigation. Secreted proteins may be integrated with biocontrol bacteria, chitosan, alginate, and other technologies to improve disease control. A chitosan–essential-oil coating reduces postharvest papaya anthracnose [196]. A pullulan coating containing biocontrol yeast and sodium chloride reduces postharvest citrus decay [40]. Separate harpin or acibenzolar-S-methyl (ASM) treatments enhance both local and systemic resistance in muskmelon [71]. Combining harpin with an antagonistic yeast further improves control of postharvest kiwifruit diseases [73].

## 6. Concluding Remarks and Perspectives

In this review, postharvest fruit immune elicitors were divided into five categories: carbohydrates, plant hormones and their functional analogs, secreted protein elicitors, inorganic compounds, and other elicitors. Together, these categories form a framework for disease control, quality maintenance, and abiotic-stress tolerance. The value of elicitors is not limited to disease prevention and control. Some treatments can also improve chilling tolerance or promote the accumulation of quality-related substances [197]. Subsequent research should comprehensively evaluate their effects on disease control, preservation, and stress tolerance. However, all elicitor categories have application limitations, and their commercial application is generally constrained by cost, formulation stability, mode of action and receptor specificity, regulatory classification, and performance under practical conditions.

The performance of carbohydrate elicitors varies with molecular weight and formulation [24,111]. Responses to inorganic compounds and hormones vary with fruit type, variety, maturity, and treatment concentration [49,65,66,89,90]. For example, melatonin can enhance tomato resistance to *B. cinerea* [65] but reduce citrus resistance to postharvest green mold [66]. Only specific receptors or host targets have been identified for some secreted protein elicitors [79,82], limiting prediction of their application range; expression, purification, and quality control further increase production costs [114,198]. Generic formulation studies indicate that proteins and peptides can be inactivated in aqueous formulations and aggregate at interfaces [191,192,193]. Formulations, packaging materials, and delivery systems should therefore be optimized for specific products [194,195]. Regulatory pathways and data requirements differ between basic substances and microbial biopesticides [199,200]. In addition to controlled experiments, disease-control efficacy, compatibility with existing treatments, residue compliance, and fruit quality should be evaluated across production areas and varieties in the field and in commercial packing houses [40,73,103].

Therefore, a phased and industrialization-oriented strategy is more realistic. (a) In the short term, candidate elicitors should be used as compatible components of existing postharvest treatment systems [71], with these systems adapted to dipping, spraying, atomization, waxing, or coating lines, and tested in combination with antagonistic yeasts [40,73], coating agents [24,40,41], or reduced fungicide doses [103]. (b) In the medium term, formulations with good persistence [139], appropriate packaging materials [194], and stable efficacy under commercial conditions [195] should be screened, and carriers and delivery systems [194,195] should be verified for specific products. (c) In the long term, receptor and host-target analyses [189,190] should guide the design of crop- and fruit-specific elicitors, and activity, stability, mechanism, and safety data should be generated and validated before registration and promotion [199,200], while the regulatory pathways for basic substances and microbial biopesticides should be clearly distinguished [199,200]. (d) Provided that efficacy and stability are maintained, the active moiety should be reduced to the minimal active peptide [114], and manufacturing costs should be reduced by fermentation or plant-derived production methods [20,80,114,198]. (e) Bifunctional carriers such as chitosan [24] and pullulan [40,41] should be selected, and the feasibility of alginate for composite formulations should be evaluated [35,36]. (f) Preharvest application regimens should be evaluated to provide protection [14,69].

## Figures and Tables

**Table 1:** Properties of different categories of postharvest fruit immune elicitors.

Category	Main Mechanism	Advantages	Application Limitations
Carbohydrate elicitors	Act as MAMP/PAMP- or DAMP-like signals and activate downstream defense responses through plasma-membrane immune receptors [23].	Derived from natural sources [23].	Effects vary with molecular weight and formulation [24,111].
Plant hormones and functional analogs	Coordinate endogenous plant hormone-signaling networks [47,48,49].	Research on the signaling network of plant endogenous hormones is relatively in-depth [47,48,49].	Hormonal crosstalk can be antagonistic, and disease-resistance effects vary among fruit species [49,65,66].
Secreted protein elicitors	Enhance the expression of fruit disease-resistance genes [77,112,113].	Highly active, effective at low concentrations, and partially thermally stable [80,114].	Most receptors remain unidentified.
Inorganic compounds	Induce secondary metabolism and exert antifungal activity [89,90].	Low cost, with readily available raw materials [89,90].	Induction is relatively weak and nonspecific [89,90].

## Data Availability

No new data were created or analyzed in this study. Data sharing is not applicable to this article.
